# Population structure and fungicide resistance in *Botrytis fragariae* from strawberry fields in the Eastern U.S.

**DOI:** 10.3389/fpls.2026.1983036

**Published:** 2026-10-19

**Authors:** Hongjin Lu, Mengjun Hu

**Affiliations:** Department of Plant Science and Landscape Architecture, University of Maryland, College Park, MD, United States

**Keywords:** *Botrytis fragariae*, fludioxonil, fungicide resistance, genomic differentiation, population genomics, strawberry gray mold

## Abstract

**Introduction:**

*Botrytis fragariae* has been frequently recovered from strawberry fields in the eastern United States and differs from *B. cinerea* in fungicide sensitivity, yet its population structure and genomic variation associated with resistance remain poorly understood.

**Methods:**

We analyzed 105 *B. fragariae* isolates from nine eastern U.S. states collected across seven sampling years to characterize population structure, fungicide-resistance profiles, and candidate genomic regions associated with fludioxonil resistance. Resistance to 10 fungicides representing seven chemical classes was evaluated, and whole-genome sequencing data were used for phylogenetic, principal component, admixture, and genome-wide differentiation analyses.

**Results:**

Phylogenetic analysis identified five clades, broadly supported by principal component analysis, while admixture analysis showed greater mixed ancestry in the two major clades than in the three smaller clades. Geographic origin and collection year did not strictly explain clade membership, with closely related isolates found across states and years, indicating broad distribution and persistence of multiple genetic backgrounds. Resistance to multiple chemical classes of fungicides, including fludioxonil, occurred more frequently in recent collections of isolates in different clades, indicating that resistance may have evolved independently. Furthermore, genome-wide weighted *F_ST_* and nucleotide-diversity scans comparing 65 fludioxonil-resistant and 40 sensitive isolates identified 15 outlier windows that merged into eight candidate genomic regions relating to the resistance. Seven of these regions contained 39 predicted genes, including genes encoding MFS/MDR transporters, cytochrome P450 proteins, transcription factors, an iron transporter, and proteins involved in membrane and redox-related processes.

**Discussion:**

Together, these findings provided a comprehensive population-genomic characterization of *B. fragariae* in Eastern U.S. strawberry fields and identified candidate genomic regions for subsequent functional investigation of fludioxonil resistance.

## 1 Introduction

Strawberry gray mold is a major fungal disease causing significant yield losses during both pre- and post-harvest stages. The fungus *Botrytis cinerea*, ranked the second most important fungal pathogen by the plant pathology community (Dean et al., 2012), was thought to be the sole cause of strawberry gray mold (Sutton, 1998). Interestingly, another species, *Botrytis fragariae*, was also revealed to cause strawberry gray mold in the U.S. and Europe in recent years (Rupp et al., 2017; Dowling et al., 2017). Although *B. fragariae* only made up 2.5% of the population examined in Germany, it comprised 35.1% of the 188 *Botrytis* isolates from multiple strawberry fields in the southeastern U.S. (Dowling et al., 2018). *B. cinerea* and *B. fragariae* were found in each of the five states examined, and both species coexisted in 11 of the 24 fields sampled and even in individual plants (Hu et al., 2018).

Previous studies have shown that *B. fragariae* differs from *B. cinerea* in several biological characteristics, including host specificity, host tissue preference, and sensitivity to fungicides (Rupp et al., 2017). While *B. cinerea* has no apparent host specificity and can infect more than 1000 plant species (Elad et al., 2016), *B. fragariae* appears to be a strawberry-specific pathogen. Based on pathogenicity assays using detached tissues/organs, *B. fragariae* was found to be slightly more aggressive on strawberry flowers, but lesion development on leaves and fruit was retarded. On non-strawberry tissues, it was shown to be incapable of causing lesions or forming very small lesions on *Gerbera* petals, apple fruit, or tomato leaves tested (Rupp et al., 2017). This phenotypic evidence suggests that *B. fragariae* may have specifically adapted to strawberries. Surveys of strawberry fields in the United States have revealed distinct fungicide resistance profiles in *B. fragariae*, with resistance detected to multiple classes of fungicides commonly used for gray mold management (Dowling et al., 2017; Lu et al., 2026). Compared with *B. cinerea*, *B. fragariae* exhibits a higher number of chemical class resistances (nCCR) and a higher frequency of resistance to the phenylpyrrole fungicide fludioxonil, while no resistance to SDHI fungicides has been reported to date (Dowling et al., 2018; Lu et al., 2026).

Fungal populations with high levels of genetic diversity, resulting from sexual reproduction and/or polycyclic life cycles, are typically at greater risk of developing fungicide resistance (McDonald and Linde, 2002; Grimmer et al., 2015). In *B. cinerea*, population studies using molecular markers and genome-wide data have shown that genetic structure is strongly associated with host origin and fungicide resistance, with fungicide selection acting as a major driver of differentiation (Mercier et al., 2021; Naegele et al., 2022; Abbey et al., 2024). Similarly, in *Plasmopara viticola*, QoI resistance arose independently in multiple genetic lineages, and resistance haplotype frequencies differed markedly among vineyard populations under local fungicide selection (Chen et al., 2007). While the population structure of *B. fragariae* remains poorly characterized, preliminary evidence suggests substantial genetic variation within the species. In a previous study, 15 haplotypes were identified among 30 isolates collected from two strawberry fields using SSR markers (Hu et al., 2018). In addition, a 3-bp insertion in *os1* was reported in U.S. but not European populations, suggesting geographic differentiation within the species (Hu et al., 2019). These studies, based on SSR markers and targeted loci, provided initial evidence of diversity in *B. fragariae*, but a genome-wide understanding of its population structure is still lacking. As a recently recognized species with distinct fungicide resistance characteristics, understanding the population structure of *B. fragariae* is important for clarifying its epidemiology and adaptation within strawberry production systems.

While fludioxonil resistance was frequently detected in *B. fragariae*, the underlying mechanisms were not fully understood. In *B. cinerea*, gain-of-function mutations in the Zn_2_Cys_6_ transcription factor gene *mrr1* can cause constitutive overexpression of the ABC transporter gene *atrB*, producing an MDR1 phenotype that includes resistance to fludioxonil (Kretschmer et al., 2009). This mechanism, however, does not explain the phenotype observed in U.S. *B. fragariae*. Although resistant isolates overexpressed *atrB*, Hu et al. (2019) found no mutations in *mrr1*; variation in the examined *atrB* upstream region, *atrB* copy number, and *os1* also failed to explain the phenotype. Genome-wide population scans could provide some insights into fungicide resistance. *F_ST_* measures genetic differentiation between resistant and sensitive populations (Weir and Cockerham, 1984), whereas nucleotide diversity (π) measures genetic variation within each population (Nei and Li, 1979). Genomic regions with elevated *F_ST_* and a high *π_S_*/*π_R_* ratio, indicating reduced diversity in resistant isolates, may therefore reflect resistance-associated selection and can be prioritized as candidate regions (Maynard Smith and Haigh, 1974). Similar approaches have identified candidate regions in other plant-pathogenic fungi: Kelly and Ward (2018) combined *F_ST_*, D_xy_, and π to identify differentiated regions under putative selection in *Fusarium graminearum*, whereas Derbyshire et al. (2019) combined population differentiation statistics with selective-sweep analysis to detect a candidate region near an MFS transporter gene in *Sclerotinia sclerotiorum*.

Given its emergence as a fungal pathogen affecting strawberry production in the United States, its distinct fungicide-resistance profile, and the unresolved genomic basis of fludioxonil resistance, a genome-wide investigation of *Botrytis fragariae* is warranted. In this study, we analyzed whole-genome sequencing data from *B. fragariae* isolates collected across multiple strawberry production regions in the United States. Using genome-wide single-nucleotide polymorphisms (SNPs), our objectives were to (i) characterize the genome-wide population structure of *B. fragariae*, (ii) examine the relationships between population structure and collection year, geographic location, mating type, and fungicide-resistance profiles, including chemical class resistance and individual fungicide-resistance phenotypes, and (iii) identify candidate genomic regions differentiated between fludioxonil-resistant and fludioxonil-sensitive isolates using *F_ST_* and nucleotide diversity (π) scans, followed by annotation of genes within these regions that may be relevant to fludioxonil resistance.

## 2 Materials and methods

### 2.1 Isolate and fungicide resistance phenotyping

A total of 105 *B. fragariae* isolates used in this study were selected from a larger collection of *Botrytis* isolates described previously (Lu et al., 2026). Isolates were mainly obtained from strawberry flowers with black centers, likely due to frost damage or a hard freeze, across nine states in the United States over seven years (Dowling et al., 2017, 2018; Hu et al., 2018).

Fungicide resistance was determined using a mycelial growth assay on fungicide-amended media at discriminatory concentrations as previously described (Fernández-Ortuño et al., 2014; Cosseboom and Hu, 2021). Ten fungicides representing seven chemical classes were tested for resistance, including boscalid, cyprodinil, fenhexamid, fludioxonil, iprodione, isofetamid, penthiopyrad, pyraclostrobin, thiophanate-methyl, and pydiflumetofen. Isolates were classified as resistant or sensitive based on the presence or absence of mycelial growth on artificial media amended with fungicides at discriminatory concentrations.

### 2.2 Whole-genome sequencing and SNP genotyping

Mycelia of each isolate were grown on cellophane-covered potato dextrose agar (PDA) (Difco, Becton, Dickinson and Company, Franklin Lakes, NJ, USA) plates for 3 days at 25 °C. Genomic DNA was extracted from 105 *B. fragariae* isolates using the E.Z.N.A.^®^ Plant & Fungal DNA Kit (Omega Bio-tek, Norcross, GA, USA) following the manufacturer’s instructions. DNA quality and integrity were assessed by agarose gel electrophoresis and NanoDrop spectrophotometry.

PCR-free paired-end libraries were constructed and sequenced on an Illumina NovaSeq 6000 platform with 100-bp paired-end reads. Sequencing was performed by CD Genomics (Shirley, NY, USA). Sequence quality was assessed using FastQC v0.11.7 (Babraham Bioinformatics, Cambridge, United Kingdom). Adapter sequences and low-quality bases were trimmed using Cutadapt v5.2 (Martin, 2011) with parameters -q 30 and -m 50, and reads shorter than 50 bp were discarded. Cleaned reads were mapped to the *B. fragariae* reference genome Bfra_R1V1 (Wu et al., 2021) using BWA-MEM v0.7.15 (Li and Durbin, 2009; Li, 2013). SAM files were converted to sorted BAM files using SAMtools v1.4.1 (Li et al., 2009).

Variant calling was performed following the GATK Best Practices workflow (Van der Auwera et al., 2013) using GATK v4.0.6.0. Variants were called using HaplotypeCaller (Poplin et al., 2017) in GVCF mode and jointly genotyped using GenotypeGVCFs. SNPs were extracted using SelectVariants and filtered using the following criteria: QD < 25.0, FS > 5.0, MQ < 55.0, MQRankSum < −0.5, ReadPosRankSum < −2.0, and SOR > 2.5. After filtering, a total of 536,687 high-quality SNPs were retained.

### 2.3 Population genomic analyses

To investigate genetic diversity and population structure, SNPs were further filtered based on minor allele frequency (MAF ≥ 0.05) and missing rate ≤ 10%. Following filtering, 401,508 SNPs were retained for downstream analyses. After linkage disequilibrium (LD) pruning using an *r²* threshold of 0.2, 11,880 SNPs were retained for population structure analyses.

Principal component analysis (PCA) was performed using PLINK v1.9 (Purcell et al., 2007; Chang et al., 2015) to visualize genetic relationships among isolates. A maximum-likelihood phylogenetic tree was constructed using IQ-TREE v3 (Nguyen et al., 2015; Wong et al., 2026) under the GTR+F+G4 substitution model with 1,000 bootstrap replicates. Population structure was inferred using ADMIXTURE v1.3.0 (Alexander et al., 2009) with K values ranging from 2 to 10, with five independent runs per K. Cross-validation (CV) error was used to evaluate model fit across K values (Alexander and Lange, 2011). Separately, genome-wide LD decay was evaluated across all 105 isolates using a random subset of 20,000 SNPs from the input VCF containing 506,528 variants. Pairwise *r²* was calculated using PLINK v1.9 (Purcell et al., 2007; Chang et al., 2015) between SNPs on the same scaffold separated by up to 500 kb (--r2 --ld--window 999999 --ld--window--kb 500 --ld--window--r2 0). Mean *r^2^* values were summarized in 5-kb physical distance bins.

### 2.4 Mating type determination

The mating type of each isolate was inferred from read coverage across mating-type-specific genomic regions, based on the known organization of MAT loci in *Botrytis* species (De Miccolis Angelini et al., 2016; Amselem et al., 2011). For MAT1–1 identification, whole-genome sequencing reads previously aligned to the *Botrytis fragariae* reference genome Bfra_R1V1 (Wu et al., 2021) using BWA-MEM (Li, 2013; Li and Durbin, 2009) were used. The genomic region corresponding to the MAT1-1–1 gene (*Bfra_000968*; NW_023500987.1:3,129,197 - 3,132,114) was extracted from the reference genome, and read depth across this region was calculated using SAMtools (Li et al., 2009). For each isolate, the proportion of sites with non-zero coverage and the mean sequencing depth across the region were calculated. Isolates with ≥80% coverage across the MAT1-1–1 region and mean depth ≥20× were classified as MAT1-1.

Because the Bfra_R1V1 assembly used here contained the MAT1–1 idiomorph but no detectable MAT1-2–1 locus, an alternative MAT1–2 reference region was generated from the *Botrytis cinerea* T4 genome. Strain T4 carries the MAT1–2 idiomorph and contains the MAT1-2–1 HMG-domain gene (Amselem et al., 2011), and the updated T4 genome assembly was described by Staats and van Kan (2012). Briefly, the MAT1-2–1 protein sequence from Sclerotinia sclerotiorum (GenBank accession AAZ83720.1) was used as a query in a tBLASTn search against the *B. cinerea* T4 genome assembly. The top-scoring homologous region in the *B. cinerea* T4 genome, together with approximately 11 kb of flanking sequence, was extracted and used as a reference for *B. fragariae* MAT1-2–1 identification. Whole-genome sequencing reads from each isolate were then independently aligned to this region using BWA-MEM, and read depth and coverage fraction across the MAT1-2–1 candidate region were calculated using SAMtools. Isolates with ≥80% coverage across the candidate MAT1-2–1 region and mean depth ≥20× were classified as MAT1-2.

### 2.5 Genome-wide scan for fludioxonil-associated differentiation

To identify genomic regions potentially associated with fludioxonil resistance, we performed a genome-wide differentiation scan using all the isolates. Starting from the genome-wide SNP dataset described above, high-quality SNPs were further filtered to retain biallelic sites with a minor allele frequency ≥ 0.05 and a missing rate ≤ 10%, resulting in 399,535 SNPs for downstream analysis.

Genetic differentiation between resistant and sensitive isolates was estimated using windowed Weir and Cockerham’s *F_ST_* in VCFtools (Weir and Cockerham, 1984; Danecek et al., 2011). Nucleotide diversity was calculated separately for sensitive and resistant isolates as *π_S_* and *π_R_*, respectively. *F_ST_*, *π_S_*, and *π_R_* were calculated in 10-kb sliding windows with a 5-kb step size, and windows containing fewer than five SNPs were excluded.

Candidate outlier windows were defined using an empirical upper-tail approach to focus on genomic intervals showing both strong differentiation and reduced nucleotide diversity in resistant isolates. Specifically, windows were retained if they fell within the top 1% of weighted *F_ST_* values and the top 5% of log_2_(*π_S_*/*π_R_*) values. Adjacent or overlapping outlier windows were merged using bedtools (Quinlan and Hall, 2010) to define candidate genomic regions. Genes overlapping candidate regions were identified using the reference genome annotation, and allele-frequency differences between resistant and sensitive isolates were calculated for SNPs located within candidate regions.

## 3 Results

### 3.1 Genome sequencing and SNP discovery

A total of 105 *Botrytis fragariae* isolates were subjected to whole-genome sequencing. After removal of low-quality reads, clean reads were aligned to the reference genome Bfra_R1V1, resulting in an average mapping rate of 98.3%, ranging from 90.4% to 99.2% across isolates. Following variant calling and quality filtering, a total of 536,687 high-quality single-nucleotide polymorphisms (SNPs) were identified genome-wide. After additional filtering based on minor allele frequency (MAF ≥ 0.05) and missing data (≤10%), 401,508 SNPs were retained for linkage disequilibrium pruning (r² = 0.2), reducing the dataset to a total of 11,880 SNPs for downstream analyses.

### 3.2 Population structure of *Botrytis fragariae*

Five clades were revealed among the 105 *B. fragariae* isolates based on maximum likelihood phylogenetic analysis, indicating a high level of genetic diversity (**Figure 1A**). Clades 1, 2, and 3, comprising a small number of isolates, seem to have diverged earlier than clades 4 and 5. Specifically, clade 1 consisted of two isolates collected in Maryland in 2021, whereas the three isolates in clade 2 were collected in North Carolina in 2016. Clade 3 comprised five isolates collected from multiple geographic locations and collection years. In contrast, most of the isolates are grouped under clades 4 and 5, with each containing 38 and 57 isolates, respectively. Similarly, isolates within these clades were collected across multiple geographic locations and sampling years. Principal component analysis (PCA) revealed clustering patterns largely consistent with the phylogenetic structure (**Figure 1B**). Isolates were color-coded according to their phylogenetic clade assignment. PC1 and PC2 explained 14.98% and 10.64% of the total genetic variation, respectively. The two major clades 4 and 5 were separated along PC1, in agreement with the phylogenetic analysis.

**Figure 1:**
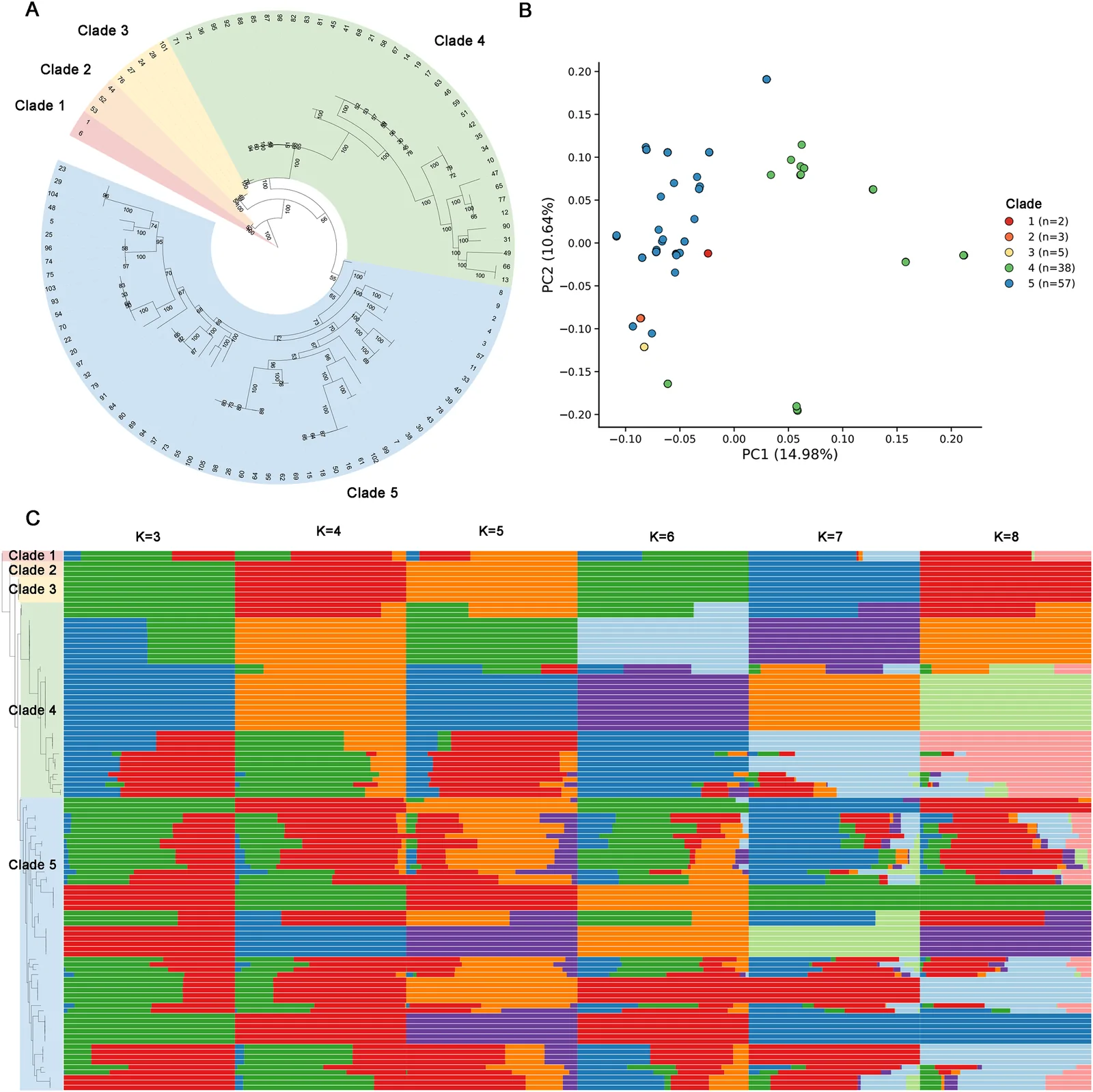
Population genomic structure of 105 *Botrytis fragariae* isolates based on genome-wide SNPs. **(A)** Maximum-likelihood phylogenetic tree showing five genetic clades. Clades are shaded with different colors. **(B)** Principal component analysis (PCA) of the isolates. Each point represents one isolate and is colored according to the clade assignment from the phylogenetic tree. PC1 and PC2 explained 14.98% and 10.64% of the genetic variation, respectively. **(C)** ADMIXTURE analysis showing inferred ancestry components from K = 3 to K = 8. Each bar represents one isolate, and colors indicate inferred ancestry components. Isolates are arranged according to the phylogenetic clade structure.

ADMIXTURE analysis was conducted to infer population history. The cross-validation error decreased continuously with increasing K values, no single optimal K value was thus identified (data not shown). Overall, it revealed population structure patterns similar to those observed in the phylogenetic and PCA analyses (**Figure 1C**). Isolates in clades 2 and 3 appeared to have relatively simple population structure patterns with mainly single ancestry components, although the number of isolates in these clades was limited. In contrast, isolates in clade 5 showed more mixed population structure patterns and appeared to have more diverse ancestry components than isolates in the other clades. Genome-wide LD decreased with increasing physical distance, with mean *r*² declining from 0.417 in the 0–5-kb distance bin to 0.056 in the 495–500-kb bin (**Supplementary Figure S1**).

### 3.3 Distribution of resistant phenotypes across time, clades, and regions

An increase in chemical class resistance (CCR) was observed over the years (**Figure 2A**). In 2012, all isolates collected were found to be either 1 or 2 CCR. During 2015, the majority of isolates were 3 CCR, followed by 2 CCR. During 2022 and 2024, the majority of the isolates were found to be 4 or 5 CCR. Moreover, CCR distributions differed among phylogenetic clades (**Figure 2B**). Populations that evolved later seemed to have increased levels of CCR compared to those that evolved earlier. For instance, Clades 1 and 2 contained only 1 or 2 CCR isolates, whereas Clade 3 contained isolates with 2 or 4 CCR. Both Clades 4 and 5 contained isolates with diverse resistance phenotypes, ranging from 1 CCR to 6 CCR (**Figure 2B**). Yet, approximately 45% and 39% of isolates in these two clades exhibited CCR ≥4. CCR also seemed to vary by region (**Table 1**). A majority of isolates (75.4%) collected from the Northeastern states, including Pennsylvania, Maryland, Delaware and Ohio, were 3–6 CCR. In contrast, only 35.4% of isolates collected from the Southeastern states, including Virginia, North Carolina, South Carolina, Georgia, and Arkansas, fell under this category. Arkansas was included in this group for descriptive purposes despite its geographic distinction from the southeastern states. Sampling was uneven across collection periods and geographic regions, with earlier collections predominantly from the Southeast and later collections from the Northeast (**Table 1**). Interestingly, this seemed to correlate with their clade distribution, where the majority of the latter were found in Clade 5.

**Figure 2:**
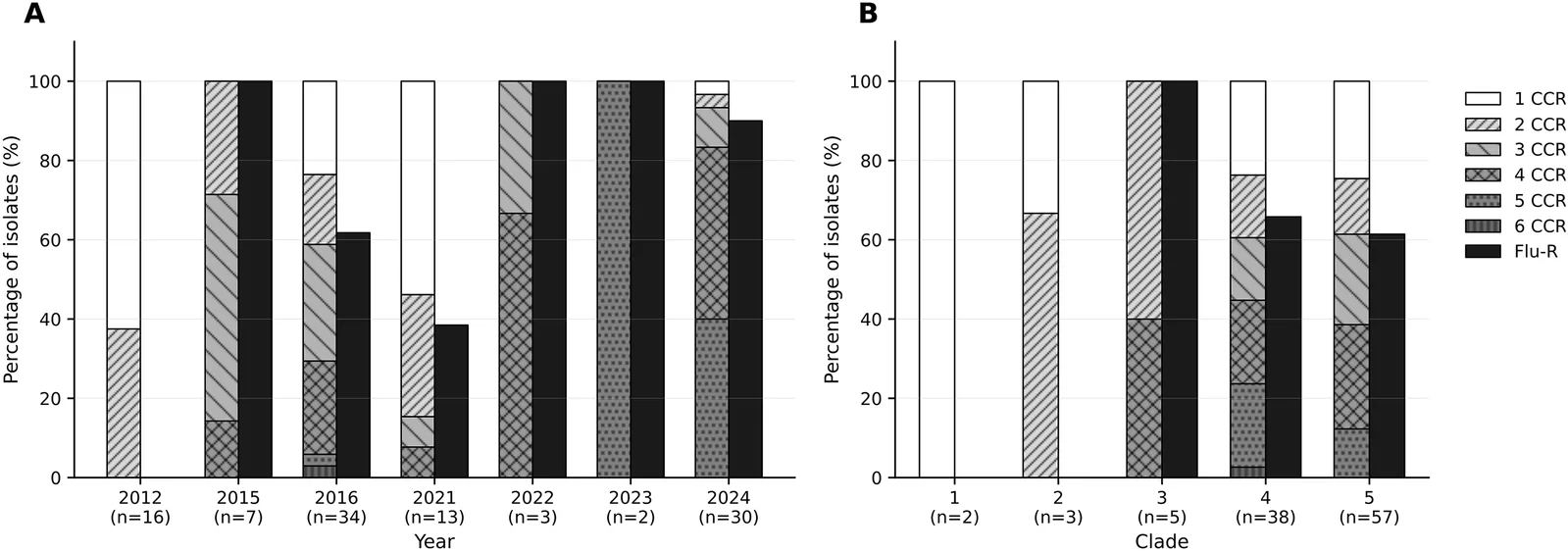
Distribution of fungicide resistance phenotypes across collection periods and phylogenetic clades in *Botrytis fragariae*. **(A)** Distribution of chemical class resistance and fludioxonil resistance across collection periods. **(B)** Distribution of chemical class resistance and fludioxonil resistance among phylogenetic clades. CCR indicates the number of fungicide chemical classes to which an isolate was resistant.

**Table 1:** Origin, resistant phenotype, mating type and clade of 105 *Botrytis fragariae* isolates used in this study.

Grouping	Category	Number of isolates	Fludioxonil resistance (%)	3–6 CCR (%)	Mating type (%)		Clade (%)				
					MAT1-1	MAT1-2	1	2	3	4	5
Region	Northeast	57	80.7	75.4	56.1	43.9	3.5	0	8.8	22.8	64.9
	Southeast	48	39.6	35.4	64.6	35.4	0	6.3	0	52.1	41.7
Time	2012–2016	57	49.1	43.9	68.4	31.6	0	5.3	0	47.4	47.4
	2021–2024	48	77.1	72.9	50	50	4.2	0	10.4	22.9	62.5

Frequencies of *B. fragariae* isolates with resistance to fludioxonil also seemed to increase over time (**Figure 2A**). For instance, all 16 isolates collected in 2012 were sensitive to fludioxonil. Among isolates collected during 2012–2016, 49.1% were resistant to fludioxonil, whereas 77.1% of isolates collected during 2021–2024 were resistant (**Table 1**). Fludioxonil resistance frequencies also differed among phylogenetic clades (**Figure 2B**). Clades 1 and 2 consisted entirely of fludioxonil-sensitive isolates, whereas all isolates in Clade 3 were resistant. In Clades 4 and 5, although both resistant and sensitive isolates were present, approximately 65.8% and 61.4% of the isolates were resistant, respectively (**Figure 2B**). Regarding its regional distribution, similar to CCR 3–6, isolates collected from the Northeast had a higher frequency (80.7%) of fludioxonil resistance than those collected from the Southeast (39.6%) (**Table 1**).

### 3.4 Frequency of mating types

Both MAT1-1–1 and MAT1-2–1 genes were detected within the *B. fragariae* population. Among the 105 isolates, 63 were classified as MAT1–1 and 42 as MAT1-2, corresponding to an approximate MAT1-1: MAT1–2 mating type ratio of 1.5:1. Geographic differences in mating-type composition were observed (**Table 1**). MAT1–1 was predominant in the Southeast, where MAT1–1 and MAT1–2 accounted for 64.6% and 35.4% of isolates, respectively, corresponding to an approximate ratio of 1.8:1. In comparison, isolates from the Northeast showed a more balanced mating-type composition, with 56.1% MAT1–1 and 43.9% MAT1-2. Mating-type frequencies also differed across collection periods. Most of the isolates collected earlier, between 2012 and 2016, were MAT1-1. In contrast, isolates collected more recently, from 2021 to 2024, showed a balanced 1:1 distribution. Overall, fludioxonil resistance was observed in 40 of 63 MAT1–1 isolates (63.5%) and 25 of 42 MAT1–2 isolates (59.5%). Both mating types were also represented across most CCRs (**Figure 3**). Counts of sensitive and resistant isolates of each mating type, tabulated separately by genetic clade, geographic origin, and collection year, are provided in **Supplementary Table S1**.

**Figure 3:**
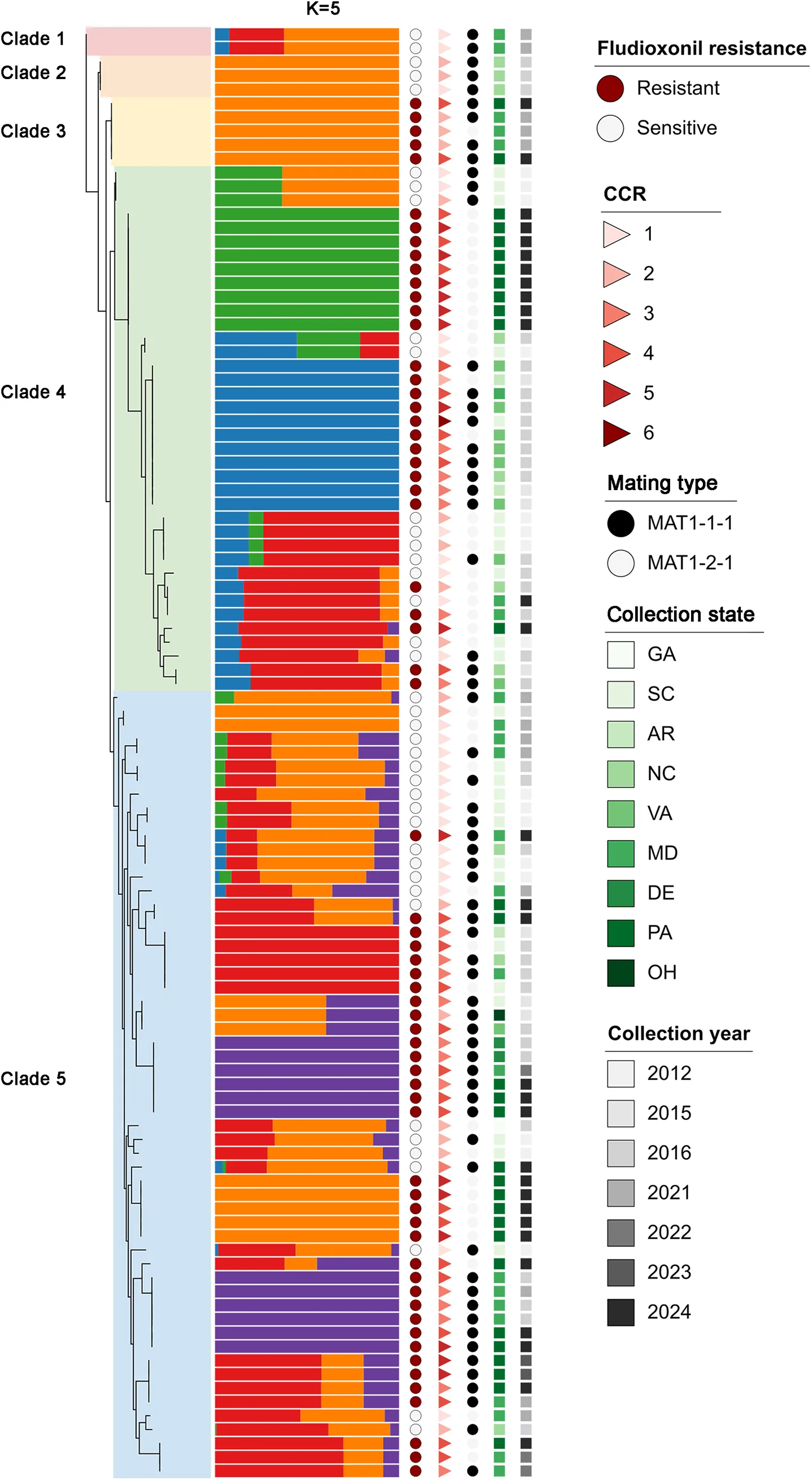
Distribution of fungicide resistance, mating type, and sampling information across *Botrytis fragariae* genetic clades. Maximum-likelihood phylogeny of 105 *B. fragariae* isolates shown together with the K = 5 ADMIXTURE ancestry profile and related information for each isolate. Each row represents one isolate and is aligned across panels. The tracks indicate fludioxonil resistance status, chemical class resistance (CCR; 1–6), mating type, collection state, and collection year.

### 3.5 Genome-wide scan highlights candidate regions

To identify genomic regions potentially associated with fludioxonil resistance, we performed genome-wide scans of genetic differentiation and nucleotide diversity between 65 fludioxonil-resistant and 40 fludioxonil-sensitive isolates using 399,535 high-quality biallelic SNPs. Weighted *F_ST_* and nucleotide diversity were calculated in 10-kb windows with 5-kb steps. Windows containing fewer than five SNPs for *F_ST_* or either nucleotide-diversity estimate were excluded, leaving 7,587 windows for analysis. The genome-wide distributions of weighted *F_ST_* and log_2_(*π_S_*/*π_R_*), where *π_S_* and *π_R_* represent nucleotide diversity in the sensitive and resistant populations, respectively, are shown in **Figures 4A, B**, and the joint distribution of the two statistics is shown in **Figure 4C**. The 99th-percentile threshold for weighted *F_ST_* was 0.1803, and the 95th-percentile threshold for log_2_(*π_S_*/*π_R_*) was 0.8640, equivalent to a *π_S_*/*π_R_* ratio of approximately 1.82. Fifteen 10-kb windows exceeded both empirical thresholds, including eight on scaffold 3, one on scaffold 4, and six on scaffold 12 (**Supplementary Table S2**). Merging overlapping or contiguous outlier windows yielded eight candidate genomic regions (**Table 2**).

**Figure 4:**
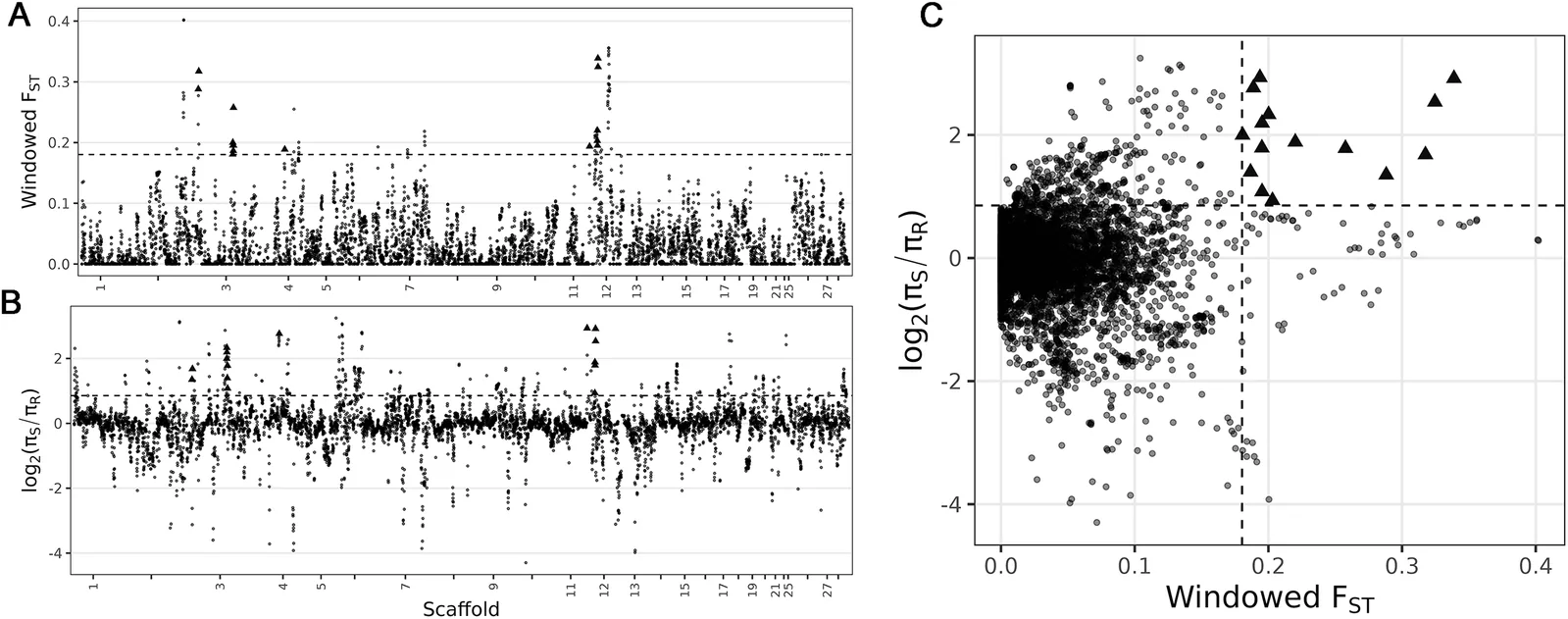
Genome-wide differentiation between fludioxonil-resistant and -sensitive *Botrytis fragariae* isolates. **(A)** Windowed *F_ST_* across scaffolds; the dashed line indicates the 99th percentile threshold (*F_ST_* = 0.1803). **(B)** Genome-wide log_2_ (*π_S_*/*π_R_*); the dashed line indicates the 95th percentile threshold (0.8640). **(C)** Joint distribution of *F_ST_* and log_2_ (*π_S_*/*π_R_*). Triangles indicate candidate windows exceeding both thresholds.

**Table 2:** Candidate genomic regions identified by combined FST and nucleotide-diversity analyses between fludioxonil-resistant and -sensitive *B. fragariae* isolates.

Region	Genomic interval	Length (kb)	No. of outlier windows	No. of unique filtered SNPs	No. of predicted genes	Max weighted *F_ST_*	Max log_2_ (*π_S_*/*π_R_*)
R1	Scaffold 3: 225,001–235,000	10	1	228	3	0.288	1.354
R2	Scaffold 3: 250,001–260,000	10	1	38	2	0.317	1.706
R3	Scaffold 3: 2,085,001–2,105,000	20	3	57	4	0.2	2.381
R4	Scaffold 3: 2,115,001–2,140,000	25	3	62	10	0.258	1.817
R5	Scaffold 4: 1,440,001–1,450,000	10	1	37	6	0.189	2.845
R6	Scaffold 12: 25,001–35,000	10	1	25	0	0.194	3.119
R7	Scaffold 12: 440,001–460,000	20	3	361	11	0.22	1.894
R8	Scaffold 12: 475,001–490,000	15	2	36	3	0.339	3.065

Candidate regions were generated by merging overlapping or contiguous outlier windows exceeding both empirical thresholds: the 99th-percentile weighted *F_ST_* threshold (0.1803) and the 95th-percentile log_2_(π_S_/π_R_) threshold (0.8640). *π_S_* and *π_R_* represent nucleotide diversity in fludioxonil-sensitive and -resistant isolates, respectively. Coordinates are shown as 1-based inclusive intervals.

The eight candidate regions ranged from 10 to 25 kb in length and contained 844 unique filtered SNPs (**Table 2**). Individual regions contained 25–361 unique filtered SNPs and were supported by one to three outlier windows. Region-level maximum weighted *F_ST_* values ranged from 0.189 to 0.339, whereas maximum log_2_(*π_S_*/*π_R_*) values ranged from 1.354 to 3.119. The highest weighted *F_ST_* occurred in the region at scaffold 12:475,001–490,000, which had a maximum weighted *F_ST_* of 0.339 and a maximum log_2_(*π_S_*/*π_R_*) of 3.065. The highest maximum log_2_(*π_S_*/*π_R_*) occurred at scaffold 12:25,001–35,000 (3.119), whereas the region at scaffold 12:440,001–460,000 contained the largest number of unique filtered SNPs (361).

Seven of the eight candidate regions contained a total of 39 predicted genes. Although the strongest region-level signals for both statistics occurred on scaffold 12, the region at scaffold 3:2,115,001–2,140,000, supported by three outlier windows, contained 10 predicted genes, several of which had annotations potentially relevant to fungicide response. These included three genes annotated as putative cytochrome P450 proteins or P450 monooxygenases (*Bfra_001820*, *Bfra_001821*, and *Bfra_001825*); three putative MFS/MDR transporter or drug-efflux genes (*Bfra_001822*, *Bfra_001823*, and *Bfra_001824*); a putative MYB DNA-binding domain-containing gene (*Bfra_001828*); a putative ent-kaurene synthase gene (*Bfra_001826*); a putative terpene synthase family gene (*Bfra_001827*); and an uncharacterized gene (*Bfra_001819*). Other candidate regions contained genes with potentially relevant annotations, including a putative siderophore iron transporter (*Bfra_001259*), a putative pH signal-transduction protein (*Bfra_001814*), a putative sphingosine N-acyltransferase (*Bfra_001816*), a putative short-chain dehydrogenase/reductase (*Bfra_002675*), a putative integral membrane protein (*Bfra_007697*), and a putative Zn_2_Cys_6_ transcriptional activator (*Bfra_007698*). Complete annotations for all 39 predicted genes are provided in **Supplementary Table S3**. Together, these regions define a focused set of genomic candidates for subsequent expression and functional analyses.

## 4 Discussion

This study provides the first population genomic analysis of *Botrytis fragariae* across major eastern strawberry-producing regions of the United States. Population structure analyses identified five phylogenetic clades, and the clustering patterns were generally supported by phylogenetic, PCA, and ADMIXTURE analyses. Although five clades were detected, most isolates belonged to Clades 4 and 5, whereas the early-diverging Clades 1, 2, and 3 were represented by only ten isolates. Despite their small sample size, Clades 1, 2, and 3 formed clear groups in both the phylogenetic tree and ADMIXTURE analysis. In contrast, isolates in Clades 4 and 5 showed more complex ancestry patterns, suggesting shared ancestry or admixture among closely related genetic backgrounds. Similar genetic diversity and population subdivision have been reported in *B. cinerea*, including high genomic variation and host-associated population structure (Atwell et al., 2015; Mercier et al., 2021; Plesken et al., 2021).

The presence of multiple phylogenetic clades suggests that the U.S. *B. fragariae* population is genetically diverse and is unlikely to represent a single recent clonal introduction. The uneven distribution of isolates among clades suggests that Clades 4 and 5 may represent more common or more successful lineages in the sampled regions. Dominance of particular *Botrytis* lineages has been reported in strawberry systems; for example, gray mold populations in German strawberry fields were dominated by *Botrytis* group S, a novel clade closely related to *B. cinerea* and *B. fabae* (Leroch et al., 2013). The high representation of Clades 4 and 5 could reflect the spread of lineages that are well adapted to strawberry production environments. Population structure can be shaped by fungicide-driven selection and host adaptation. For example, *B. cinerea* populations infecting small-fruit hosts can rapidly respond to fungicide selection in agricultural environments (Kozhar et al., 2020), and genetically isolated *B. cinerea* populations showing distinct host adaptation have also been reported (Plesken et al., 2021). Therefore, the dominance of Clades 4 and 5 in this study may reflect biological prevalence, adaptation to production conditions, or repeated movement through planting material.

Despite the presence of distinct phylogenetic clades, a lack of geographic structure within the U.S. *B. fragariae* population was revealed. Isolates from different regions often clustered together rather than forming clear region-specific groups. This pattern suggests that geographic distance alone is not the main factor shaping the observed population structure. Similar weak location-associated differentiation has been reported in *B. cinerea* populations. In Michigan vineyards, for example, population structure was associated more strongly with fungicide resistance and year than with sampling location (Naegele et al., 2021). One likely explanation is the frequent movement of strawberry planting material. Annual plasticulture systems commonly depend on new nursery-produced transplants each season, and these plants may be moved over long distances before being planted in fruit production fields (Santos et al., 2012; Oliveira et al., 2017). Most isolates in the present study were collected from plasticulture production systems, where annual transplanting could repeatedly introduce pathogen genotypes from outside the local region. Previous studies have shown that strawberry transplants can carry latent *Botrytis* infections without visible symptoms and may serve as an important source of primary inoculum in commercial strawberry fields (Oliveira et al., 2017). *Botrytis* cryptic species have also been detected in strawberry nursery transplants and commercial fields in the eastern United States (Amiri et al., 2018). In addition, transplants may already carry fungicide-resistant isolates because of fungicide exposure in nursery production systems (Oliveira et al., 2017). Therefore, movement of infected or latently infected planting material could introduce related pathogen genotypes into geographically distant fields.

Local dispersal after field establishment may further reduce geographic differentiation. *Botrytis* species produce airborne conidia, and airborne *B. cinerea* conidial concentrations have been linked with gray mold incidence in strawberry flowers and fruits (Blanco et al., 2006; Williamson et al., 2007). Thus, dispersal of *B. fragariae* may involve two connected processes: long-distance movement through infected planting material, followed by local spread through airborne conidia within and among nearby fields. This combination of human-mediated and natural dispersal could explain why isolates from different regions were genetically mixed. However, the present study did not include detailed nursery-origin information for each isolate, and sample sizes differed among regions and years. These factors may reduce the ability to detect geographic structure. Future studies linking nursery and field isolates to transplant origins could clarify the drivers of such geographic structure.

Fungicide resistance was detected across multiple phylogenetic clades, indicating that resistance is not confined to a single genetic lineage. Resistant isolates, including fludioxonil-resistant isolates and those displaying resistance to multiple chemical classes (e.g., 4 or 5 CCR), were distributed across clades rather than clustering in the same clades. This pattern is consistent with repeated emergence or selection of resistance on different genetic backgrounds, rather than the spread of a single resistant lineage. Similar independent emergence of resistance to multiple fungicide classes has been reported in *B. cinerea* populations from small-fruit systems (Fernández-Ortuño et al., 2015).

Resistance patterns also changed over time. More recent isolates generally showed higher CCR and a greater frequency of fludioxonil resistance than isolates collected in earlier years. This pattern is consistent with the progressive enrichment of resistant phenotypes under sustained fungicide selection over successive growing seasons (Hu et al., 2016; Rupp et al., 2017). For example, fenhexamid resistance increased progressively in *B. cinerea* populations from Florida strawberry fields sampled between 2005 and 2013 (Amiri and Peres, 2014), while fludioxonil resistance in a German raspberry field increased from below 10% in 2010 to over 85% in 2015 (Rupp et al., 2017). These findings support cumulative selection from repeated fungicide use as a likely contributor to the higher resistance observed in our more recent collections, although the effects of collection period and geographic origin cannot be separated due to uneven sampling. Fludioxonil was first registered in the United States in 1995, later than most of the fungicides commonly used for strawberry disease management. While resistance to fludioxonil has been detected in *Botrytis* isolates, its frequency was found to be lower than that of many other fungicides (Fernández-Ortuño et al., 2014). A previous study has demonstrated that multifungicide resistance patterns did not evolve randomly in populations. Instead, strains resistant to newer fungicides are likely built on the backbone of resistance to older fungicides, following a stepwise accumulation (Li et al., 2014). Moreover, *Botrytis* isolates carrying multiple resistance traits can be indirectly selected when fungicide programs repeatedly use or rotate with different fungicide classes to which the isolates are resistant, termed “selection by association” (Hu et al., 2016). It is therefore expected that many isolates resistant to fludioxonil may have already developed resistance to others. Repeated fungicide applications and reliance on fludioxonil could then have favored the selection for multiresistant phenotypes over the years. Although resistant phenotypes may incur fitness costs based on lab experiments (Chen et al., 2016), comparable or even improved fitness was observed in Botrytis isolates with multifungicide resistance under field conditions (Cosseboom et al., 2020). The predominance of Clades 4 and 5 in our collection may reflect fitness costs that are limited, compensated, or outweighed by the benefits of resistance under fungicide selection, although fitness was not directly assessed in this study.

Although *B. fragariae* was formally described only recently, it has been detected in strawberry fields in both Germany and the U.S. (Rupp et al., 2017; Dowling et al., 2017). A previous study showed that *B. fragariae* comprised 35.1% of the 188 *Botrytis* isolates from multiple strawberry fields in the southeastern U.S. (Dowling et al., 2018). Further, resistance to multiple chemical classes of fungicides has also been frequently detected in *B. fragariae*, suggesting that this pathogen may have been evolving in strawberry fields for a long time before it was recognized as a species.

Mating-type analysis showed that both MAT1–1 and MAT1–2 occurred in the U.S. *B. fragariae* population, but MAT1–1 was more common overall. The distribution was not uniform across sampling periods, regions, or clades. Earlier isolates showed a stronger bias toward MAT1-1, whereas later isolates had an approximately balanced MAT1-1: MAT1–2 ratio. Northeastern populations also showed an approximately 1:1 ratio, while southeastern populations contained more MAT1–1 isolates. At the clade level, Clades 1–3 were mainly composed of MAT1–1 isolates, whereas the dominant Clades 4 and 5 included substantial numbers of both mating types. Comparisons of mating type and fludioxonil sensitivity were descriptive and do not establish whether mating type is associated with resistance independently of genetic clade, geographic origin, and collection year.

The shift toward a more balanced ratio in later collections, together with the mixed mating-type composition of Clades 4 and 5, may increase the opportunity for outcrossing and recombination. Sexual reproduction has been reported in *B. cinerea* and has also been suggested as a possible source of genetic diversity in *B. fragariae* populations from Germany (Váczy et al., 2008; Rupp et al., 2017; Rodenburg et al., 2018). The more balanced ratio in later collections could also reflect temporal replacement or differential expansion of clonal lineages carrying different mating types, rather than increased sexual reproduction. However, mating-type distribution alone is not sufficient to determine reproductive mode. In *Botrytis*, successful genotypes can spread through repeated asexual reproduction by conidia, while sclerotia support survival under unfavorable conditions and may also contribute to sexual reproduction through apothecia formation (Canessa et al., 2013; Rodenburg et al., 2018). The higher frequency of MAT1–1 in southeastern populations may reflect expansion of successful asexual lineages under longer and more favorable disease seasons, although transplant movement and uneven sampling could also have contributed to this pattern. Similar studies in *B. cinerea* showed that populations may reproduce mainly asexually even when both mating types are present or when ratios are close to 1:1 (Wessels et al., 2013, 2016). Genome-wide LD decay provided additional evidence consistent with historical recombination in the U.S. *B. fragariae* collection. Mean *r²* decreased from 0.417 in the 0–5-kb bin to 0.056 in the 495–500-kb bin, with a gradual decline across tens to hundreds of kilobases (**Supplementary Figure S1**). Atwell et al. (2018) reported that mean *r²* in a *B. cinerea* collection decreased by approximately 50% within 1 kb, while Mercier et al. (2021) reported LD half-decay distances of 3.1–16.8 kb among French *B. cinerea* populations. The decline in LD of *B. fragariae* thus seemed much lower compared to those studies, suggesting limited sexual recombination in the population examined. Further tests and observations of sexual structures would validate the role and frequency of sexual reproduction in U.S. *B. fragariae*. Future comparisons with European isolates would also help clarify the origin, movement, and reproductive history of major lineages (Rupp et al., 2017).

The joint *F_ST_* and *π_S_*/*π_R_* scan identified eight genomic regions that were differentiated between fludioxonil-resistant and sensitive isolates and showed reduced nucleotide diversity in the resistant group. Similar approaches combining population differentiation with reduced diversity or selective-sweep statistics have been used to prioritize candidate regions in *Fusarium graminearum*, *Zymoseptoria tritici*, *Microbotryum* spp., and *Sclerotinia sclerotiorum* (Kelly and Ward, 2018; Hartmann et al., 2018; Badouin et al., 2017; Derbyshire et al., 2019). These eight regions therefore provide an initial genome-wide framework for investigating fludioxonil resistance in *B. fragariae*. The scaffold 3:2,115,001–2,140,000 interval contained a compact set of 10 genes with predicted functions in transport, metabolism, and transcriptional regulation. The three putative MFS/MDR transporter genes (*Bfra_001822*, *Bfra_001823*, and *Bfra_001824*) are biologically plausible candidates because enhanced fungicide efflux is a well-known resistance mechanism (Yin et al., 2023). In *B. cinerea*, the MDR1 and MDR2 phenotypes are driven by constitutive overexpression of the ABC transporter gene *atrB* and the MFS transporter gene *mfsM2*, respectively (Kretschmer et al., 2009). The three cytochrome P450 genes (*Bfra_001820*, *Bfra_001821*, and *Bfra_001825*) may contribute through fungicide metabolism or detoxification; P450-mediated resistance to multiple fungicides with different modes of action has been experimentally demonstrated in *Rhizoctonia solani* (Cheng et al., 2022). The MYB-domain gene *Bfra_001828* represents an additional regulatory candidate. In *Fusarium graminearum*, the MYB-like transcription factor MYT3 influences nitrogen metabolism and trichothecene biosynthesis, supporting a broader role for fungal MYB proteins in metabolic regulation (Kim et al., 2014).

Additionally, *Bfra_007698* on the 12:440,001–460,000 interval encodes a putative Zn_2_Cys_6_ transcriptional activator, belonging to the same transcription-factor family as *mrr1*. Members of this family regulate fungicide-response pathways in other plant-pathogenic fungi. For example, the transcription factor FgAtrR regulates the expression of the *FgCYP51A* gene and multiple ABC transporter genes and contributes to azole resistance in *F. graminearum*, whereas mutations in the *MoIRR* gene confer isoprothiolane resistance in *M. oryzae*; *MoIRR*-deficient strains also show altered expression of genes in the Hog1 MAPK pathway and increased sensitivity to fludioxonil (Zhao et al., 2022; Wang et al., 2018, 2022). Other candidate genes across the eight regions were associated with iron and oxidative-stress homeostasis, membrane and sphingolipid organization, cellular signaling, and redox metabolism. These processes have previously been linked to stress tolerance, membrane permeability, and fungicide sensitivity in other fungi (Hof et al., 2009; Wang et al., 2023). Future work should characterize genetic and expression variation across these regions, and functionally validate key candidates.

Overall, this study provides the first genome-wide assessment of population structure, fungicide resistance, and mating-type distribution in U.S. *Botrytis fragariae* populations. The detection of multiple clades, weak geographic structure, resistance across different genetic backgrounds, and both mating types indicates that this pathogen is genetically diverse and may be shaped by fungicide selection, pathogen movement, and potential recombination. In addition, the genome-wide differentiation and nucleotide-diversity scan identified candidate genomic regions potentially associated with fludioxonil resistance, providing an initial framework for investigating its unresolved genetic basis. These findings provide a foundation for tracking the spread and evolution of *B. fragariae*, for improved disease and resistance management in the long term.

## Data Availability

The datasets presented in this study can be found in online repositories. The names of the repository/repositories and accession number(s) can be found below: https://www.ncbi.nlm.nih.gov/, PRJNA1517687.
