# Supplementary material for: Population structure and fungicide resistance in Botrytis fragariae from strawberry fields in the Eastern U.S
Source: Front Plant Sci. 2026 Oct 19;17:1983036. doi: 10.3389/fpls.2026.1983036 (PMC13724626; doi:10.3389/fpls.2026.1983036)
Supplement: Supplementary file 1 [file DataSheet1.docx]

**Supplementary Material**

**Figure S1. Genome-wide linkage disequilibrium (LD) decay in 105 *Botrytis fragariae* isolates.** Pairwise *r*^2^ was calculated using PLINK v1.9 for a random subset of 20,000 SNPs. Only SNP pairs on the same scaffold and separated by up to 500 kb were considered. The curve connects mean *r*^2^ values in 5-kb physical-distance bins, plotted at bin midpoints.

**Table S1.** Distribution of fludioxonil sensitivity by mating type, genetic clade, geographic origin, and collection year in the U.S. *Botrytis fragariae* population.

| **Grouping factor** | **Category** | **Total number** | **MAT1-1** | | | **MAT1-2** | | |
| --- | --- | --- | --- | --- | --- | --- | --- | --- |
|  |  |  | **Sensitive n** | **Resistant n** | **Resistant (%)** | **Sensitive n** | **Resistant n** | **Resistant (%)** |
| Overall | All isolates | 105 | 23 | 40 | 63.50% | 17 | 25 | 59.50% |
| Genetic clade | Clade 1 | 2 | 2 | 0 | 0.00% | 0 | 0 | — |
|  | Clade 2 | 3 | 3 | 0 | 0.00% | 0 | 0 | — |
|  | Clade 3 | 5 | 0 | 4 | 100.00% | 0 | 1 | 100.00% |
|  | Clade 4 | 38 | 5 | 11 | 68.80% | 8 | 14 | 63.60% |
|  | Clade 5 | 57 | 13 | 25 | 65.80% | 9 | 10 | 52.60% |
| Geographic region | Northeast | 57 | 6 | 26 | 81.30% | 5 | 20 | 80.00% |
|  | Southeast | 48 | 17 | 14 | 45.20% | 12 | 5 | 29.40% |
| State | AR | 3 | 0 | 2 | 100.00% | 0 | 1 | 100.00% |
|  | DE | 2 | 0 | 2 | 100.00% | 0 | 0 | — |
|  | GA | 1 | 0 | 0 | — | 1 | 0 | 0.00% |
|  | MD | 24 | 4 | 12 | 75.00% | 5 | 3 | 37.50% |
|  | NC | 10 | 5 | 3 | 37.50% | 1 | 1 | 50.00% |
|  | OH | 1 | 0 | 1 | 100.00% | 0 | 0 | — |
|  | PA | 30 | 2 | 11 | 84.60% | 0 | 17 | 100.00% |
|  | SC | 25 | 11 | 2 | 15.40% | 10 | 2 | 16.70% |
|  | VA | 9 | 1 | 7 | 87.50% | 0 | 1 | 100.00% |
| Collection year | 2012 | 16 | 9 | 0 | 0.00% | 7 | 0 | 0.00% |
|  | 2015 | 7 | 0 | 6 | 100.00% | 0 | 1 | 100.00% |
|  | 2016 | 34 | 8 | 16 | 66.70% | 5 | 5 | 50.00% |
|  | 2021 | 13 | 4 | 4 | 50.00% | 4 | 1 | 20.00% |
|  | 2022 | 3 | 0 | 2 | 100.00% | 0 | 1 | 100.00% |
|  | 2023 | 2 | 0 | 2 | 100.00% | 0 | 0 | — |
|  | 2024 | 30 | 2 | 10 | 83.30% | 1 | 17 | 94.40% |

**Table S2.** Outlier genomic windows exceeding both empirical thresholds for genetic differentiation and nucleotide diversity between fludioxonil-resistant and -sensitive *Botrytis fragariae* isolates.

| **Scaffold** | **Window start** | **Window end** | **No. of SNPs for F_ST_** | **Weighted F_ST_** | **Mean F_ST_** | **No. of SNPs (S)** | **π_S_** | **No. of SNPs (R)** | **π_R_** | **π_S_/π_R_** | **log₂(π_S_/π_R_)** |
| --- | --- | --- | --- | --- | --- | --- | --- | --- | --- | --- | --- |
| 3 | 225,001 | 235,000 | 228 | 0.288148 | 0.280849 | 210 | 0.0105122 | 228 | 0.00411255 | 2.556078 | 1.353932 |
| 3 | 250,001 | 260,000 | 38 | 0.317480 | 0.311003 | 37 | 0.0018338 | 37 | 0.0005622 | 3.261829 | 1.705681 |
| 3 | 2,085,001 | 2,095,000 | 46 | 0.180660 | 0.175623 | 45 | 0.00199962 | 46 | 0.000489445 | 4.085485 | 2.030507 |
| 3 | 2,090,001 | 2,100,000 | 39 | 0.200178 | 0.197572 | 39 | 0.00180291 | 39 | 0.000346047 | 5.210015 | 2.381287 |
| 3 | 2,095,001 | 2,105,000 | 11 | 0.195285 | 0.184462 | 11 | 0.000486456 | 11 | 9.47406e-05 | 5.134610 | 2.360255 |
| 3 | 2,115,001 | 2,125,000 | 5 | 0.186646 | 0.137310 | 5 | 0.000176583 | 5 | 5.78175e-05 | 3.054145 | 1.610768 |
| 3 | 2,125,001 | 2,135,000 | 33 | 0.257640 | 0.246213 | 33 | 0.00154696 | 33 | 0.000439173 | 3.522439 | 1.816575 |
| 3 | 2,130,001 | 2,140,000 | 55 | 0.195285 | 0.166762 | 55 | 0.00205418 | 55 | 0.000964985 | 2.128717 | 1.089984 |
| 4 | 1,440,001 | 1,450,000 | 37 | 0.188786 | 0.186005 | 37 | 0.00159835 | 37 | 0.000222397 | 7.186922 | 2.845374 |
| 12 | 25,001 | 35,000 | 25 | 0.193660 | 0.192175 | 24 | 0.000817 | 6 | 9.4e-05 | 8.685396 | 3.118592 |
| 12 | 440,001 | 450,000 | 282 | 0.203041 | 0.204883 | 281 | 0.013152 | 232 | 0.006867 | 1.915217 | 0.937508 |
| 12 | 445,001 | 455,000 | 156 | 0.220117 | 0.224639 | 156 | 0.006998 | 93 | 0.001883 | 3.716878 | 1.894091 |
| 12 | 450,001 | 460,000 | 79 | 0.195140 | 0.197035 | 79 | 0.003514 | 61 | 0.001006 | 3.494198 | 1.804961 |
| 12 | 475,001 | 485,000 | 20 | 0.338820 | 0.337813 | 20 | 0.001006 | 20 | 0.00012 | 8.369006 | 3.065056 |
| 12 | 480,001 | 490,000 | 35 | 0.324555 | 0.322626 | 35 | 0.001688 | 35 | 0.000279 | 6.051976 | 2.597406 |

**Note.** Windows exceeded both the 99th-percentile weighted *F_ST_* threshold (0.1803) and the 95th-percentile log₂(*π_S_/π_R_*) threshold (0.8640). *π_S_* and *π_R_* represent nucleotide diversity in fludioxonil-sensitive and -resistant isolates, respectively. Coordinates are shown as 1-based inclusive intervals.

**Table S3.** Predicted genes and functional annotations within candidate genomic regions identified by the genome-wide differentiation and nucleotide-diversity scan.

| **Region** | **Scaffold** | **Scaffold accession** | **Region start** | **Region end** | **Gene ID** | **Predicted annotation** |
| --- | --- | --- | --- | --- | --- | --- |
| R1 | 3 | NW_023500988.1 | 225,001 | 235,000 | *Bfra_001248* | Uncharacterized protein |
| R1 | 3 | NW_023500988.1 | 225,001 | 235,000 | *Bfra_001249* | Putative polysaccharide deacetylase protein |
| R1 | 3 | NW_023500988.1 | 225,001 | 235,000 | *Bfra_001250* | Uncharacterized protein |
| R2 | 3 | NW_023500988.1 | 250,001 | 260,000 | *Bfra_001259* | Putative siderophore iron transporter MirB protein |
| R2 | 3 | NW_023500988.1 | 250,001 | 260,000 | *Bfra_001261* | Uncharacterized protein |
| R3 | 3 | NW_023500988.1 | 2,085,001 | 2,105,000 | *Bfra_001813* | Putative SH3-domain protein |
| R3 | 3 | NW_023500988.1 | 2,085,001 | 2,105,000 | *Bfra_001814* | Putative pH signal-transduction protein |
| R3 | 3 | NW_023500988.1 | 2,085,001 | 2,105,000 | *Bfra_001815* | Uncharacterized protein |
| R3 | 3 | NW_023500988.1 | 2,085,001 | 2,105,000 | *Bfra_001816* | Putative sphingosine N-acyltransferase Lac1 protein |
| R4 | 3 | NW_023500988.1 | 2,115,001 | 2,140,000 | *Bfra_001819* | Uncharacterized protein |
| R4 | 3 | NW_023500988.1 | 2,115,001 | 2,140,000 | *Bfra_001820* | Putative cytochrome P450 protein |
| R4 | 3 | NW_023500988.1 | 2,115,001 | 2,140,000 | *Bfra_001821* | Putative cytochrome P450 protein |
| R4 | 3 | NW_023500988.1 | 2,115,001 | 2,140,000 | *Bfra_001822* | Putative MFS/MDR transporter protein |
| R4 | 3 | NW_023500988.1 | 2,115,001 | 2,140,000 | *Bfra_001823* | Putative MFS multidrug transporter protein |
| R4 | 3 | NW_023500988.1 | 2,115,001 | 2,140,000 | *Bfra_001824* | Putative MFS drug-efflux protein |
| R4 | 3 | NW_023500988.1 | 2,115,001 | 2,140,000 | *Bfra_001825* | Putative cytochrome P450 monooxygenase protein |
| R4 | 3 | NW_023500988.1 | 2,115,001 | 2,140,000 | *Bfra_001826* | Putative ent-kaurene synthase protein |
| R4 | 3 | NW_023500988.1 | 2,115,001 | 2,140,000 | *Bfra_001827* | Putative terpene synthase family protein |
| R4 | 3 | NW_023500988.1 | 2,115,001 | 2,140,000 | *Bfra_001828* | Putative MYB DNA-binding domain-containing protein |

**Table S3. Continued.**

| **Region** | **Scaffold** | **Scaffold accession** | **Region start** | **Region end** | **Gene ID** | **Predicted annotation** |
| --- | --- | --- | --- | --- | --- | --- |
| R5 | 4 | NW_023500989.1 | 1,440,001 | 1,450,000 | *Bfra_002673* | Putative exonuclease V protein |
| R5 | 4 | NW_023500989.1 | 1,440,001 | 1,450,000 | *Bfra_002674* | Uncharacterized protein |
| R5 | 4 | NW_023500989.1 | 1,440,001 | 1,450,000 | *Bfra_002675* | Putative short-chain dehydrogenase/reductase (SDR) protein |
| R5 | 4 | NW_023500989.1 | 1,440,001 | 1,450,000 | *Bfra_002676* | Putative carbohydrate-binding module family 1 protein |
| R5 | 4 | NW_023500989.1 | 1,440,001 | 1,450,000 | *Bfra_002677* | Putative glycosyltransferase family 22 protein |
| R5 | 4 | NW_023500989.1 | 1,440,001 | 1,450,000 | *Bfra_002678* | Putative snoRNP assembly factor protein |
| R7 | 12 | NW_023500997.1 | 440,001 | 460,000 | *Bfra_007689* | Uncharacterized protein |
| R7 | 12 | NW_023500997.1 | 440,001 | 460,000 | *Bfra_007690* | Uncharacterized protein |
| R7 | 12 | NW_023500997.1 | 440,001 | 460,000 | *Bfra_007691* | Putative thaumatin-like protein |
| R7 | 12 | NW_023500997.1 | 440,001 | 460,000 | *Bfra_007692* | Putative DNA repair and transcription factor protein |
| R7 | 12 | NW_023500997.1 | 440,001 | 460,000 | *Bfra_007693* | Putative RING-2 protein |
| R7 | 12 | NW_023500997.1 | 440,001 | 460,000 | *Bfra_007694* | Putative dihydroorotate oxidase protein |
| R7 | 12 | NW_023500997.1 | 440,001 | 460,000 | *Bfra_007695* | Uncharacterized protein |
| R7 | 12 | NW_023500997.1 | 440,001 | 460,000 | *Bfra_007696ia* | Uncharacterized protein |
| R7 | 12 | NW_023500997.1 | 440,001 | 460,000 | *Bfra_007696ib* | Putative domain-containing protein |
| R7 | 12 | NW_023500997.1 | 440,001 | 460,000 | *Bfra_007697* | Putative integral membrane protein |
| R7 | 12 | NW_023500997.1 | 440,001 | 460,000 | *Bfra_007698* | Putative Zn(II)₂Cys₆ transcriptional activator protein |
| R8 | 12 | NW_023500997.1 | 475,001 | 490,000 | *Bfra_007707* | Putative GLE1-like protein |
| R8 | 12 | NW_023500997.1 | 475,001 | 490,000 | *Bfra_007708* | Putative RNB-domain-containing protein |
| R8 | 12 | NW_023500997.1 | 475,001 | 490,000 | *Bfra_007709* | Putative twin-arginine translocation pathway signal protein |

**Note.** Region IDs correspond to Table 2. Region R6 (Scaffold 12:25,001–35,000) contained no annotated protein-coding gene and is therefore not listed in this gene table. Coordinates are shown as 1-based inclusive intervals.
